# BCG Therapy of Bladder Cancer Stimulates a Prolonged Release of the Chemoattractant CXCL10 (IP10) in Patient Urine

**DOI:** 10.3390/cancers11070940

**Published:** 2019-07-04

**Authors:** Omodele Ashiru, Gloria Esteso, Eva M. García‐Cuesta, Eva Castellano, Celia Samba, Eva Escudero-López, Sheila López‐Cobo, Mario Álvarez-Maestro, Ana Linares, Mei M. Ho, Asier Leibar, Luis Martínez‐Piñeiro, Mar Valés‐Gómez

**Affiliations:** 1 Division of Bacteriology, Medicines and Healthcare products Regulatory Agency-National Institute for Biological Standards and Control (MHRA-NIBSC), Blanche Lane, South Mimms, Potters Bar, Hertfordshire EN6 3QG, UK; 2 Department of Immunology and Oncology, National Centre for Biotechnology, Spanish National Research Council, 28049 Madrid, Spain; 3 Hospital La Paz Institute for Health Research (IdiPAZ), Autonomous University of Madrid, 28046 Madrid, Spain; 4 Urology Unit, Infanta Sofía Hospital, 28703 Madrid, Spain; 5 Servicio de Urología, Hospital La Paz Institute for Health Research (IdiPAZ), 28046 Madrid, Spain

**Keywords:** cytokines, chemokines, NMIBC, BCG, biomarker

## Abstract

*Background:* Intra-vesical instillation of *Bacille Calmette–Guérin* (BCG), an attenuated strain of *Mycobacterium bovis*, is an effective therapy for high-grade non-muscle invasive bladder cancer (NMIBC), which provokes a local immune response resulting in 70% of patients free of relapse after three years. Because non-responder patients usually have a bad prognosis, the early identification of treatment failure is crucial. We hypothesized that, if an effective immune response was taking place in the bladder, soluble factors would be released to the urine many days after BCG instillations. *Methods:* An extensive panel of cytokines and chemokines released into the urine seven days after every BCG instillation was screened in a cohort of NMIBC patients over three years. *Results:* The determinations of the urinary concentrations of cytokines, chemokines, and creatinine showed that increasing concentrations of C-X-C motif chemokine 10 (CXCL10) also known as interferon-inducible protein 10 (IP10) could be detected during the six-week induction cycle of BCG-treated patients released into the urine by CD14^+^ cells. In vitro, CXCL10 facilitated the recruitment of effector immune cells after the BCG-mediated upregulation of CXCR3 in both T- and natural killer (NK)-cells. *Conclusions:* The high concentrations of chemokine detected one week after the encounter with mycobacteria suggest that the CXCL10 axis might be related to the intensity of the immune anti-tumor response.

## 1. Introduction

The intravesical instillation of *Bacille Calmette–Guérin* (BCG), an attenuated strain of *Mycobacterium bovis*, has been known to be an effective treatment for high-grade non-muscle invasive bladder cancer (NMIBC) for several decades, and, in general, is more effective than chemotherapy [1]. However, not all patients respond well, so whilst around 70% of patients remain relapse-free for at least three years post treatment, others abandon treatment because of local adverse effects, mycobacterial spread, or tumor recurrence [2]. Non-responder patients have a poor prognosis and often require more aggressive treatment, such as radical cystectomy. Thus, there is a need to identify these patients early in order to offer alternative therapies. Currently, there are no predictive methods for the identification of non-responder NMIBC patients, other than the identification of recurrence by biopsy [3,4,5]. Clinico-pathological variables, including tumor grade, stage, size, number, and the presence/absence of previous recurrences, remain the only prognostic predictors used in the clinics. Therefore, although BCG instillation is successful for NMIBC treatment, further study of the mechanisms underlying the efficacy of this immunotherapy is needed in order to define the biomarkers that could allow for the early identification of non-responding patients [2].

We have reported that the in vitro exposure of peripheral mononuclear blood cells (PBMCs) to BCG provokes the activation of an unusual cytotoxic CD56^bright^ natural killer (NK) cell population that responds very efficiently against bladder cancer cells [6]. The BCG-priming of CD56^bright^ NK cells depended on the release of soluble factors that occurred in several waves over a week [6]. Subsequently, other groups have also described the expansion of CD56^bright^ NK cells with anti-tumor activities, and associated this with interleukin 15 (IL-15) treatment [7]. Cytokines are good candidates for following the immune response in patients, and, thus, they have been studied in the urine of NMIBC patients 24–48 h after instillations with BCG. In general, early after instillation, various pro-inflammatory cytokines are found in urine, with tumor necrosis factor (TNF)α, IL-6, IL-1β, and IL-8 being predominant [8,9,10]. In this type of study, elevated urinary Vascular Endothelial Growth Factor (VEGF) appeared as an independent predictor of NMIBC [11]. Based on the concentration of soluble factors in combination with the clinical data, a nomogram to predict NMIBC recurrence (CyPRIT) has been proposed [12,13]. Although several of these potential predictive markers appear in different published studies, urine samples were always collected in the first 4–48 h after BCG instillation, when a considerable degree of acute inflammation is still ongoing. We hypothesized that the analysis of the soluble factors still being released into urine after longer times of BCG instillation could provide novel information on aspects of the prolonged immune response taking place in each patient at the site of the tumor. Thus, we initiated a study in which we quantified the cytokines in the urine samples collected seven days after instillation, considerably later than the other published studies. Analysis at this time point had the additional benefit of being more convenient for patients, as this time coincided with the patients’ weekly visit to the clinic for follow-up instillations.

Here, we evaluated the cytokine amounts in the urine samples collected from BCG-treated NMIBC patients one week after BCG instillation, along several cycles of treatment. This exploratory study was designed to obtain a high number of samples along the treatment course, and a wide range of cytokines and chemokines, similar to those previously tested in vitro [6] was assayed. Interestingly, the C-X-C motif chemokine 10 (CXCL10), also known as interferon -induced protein 10 (IP10), showed a consistent pattern of stepwise increases in all NMIBC patients that responded to BCG therapy. However, patients with very high concentrations of CXCL10, especially when accompanied by increased IL-6 and IL-8, suffered adverse effects like cystitis. The intracellular staining of cells released to the urine identified CD14 myeloid cells as producers of CXCL10. BCG-treated T and NK cells upregulate the receptor for CXCL10, CXCR3, and, in turn, they migrate more efficiently towards CXCL10.

In aggregate, these data, especially the prolonged increase in IP10 production observed seven days after the instillation, suggests that CXCL10, along with other pro-inflammatory cytokines, might be a useful biomarker for the classification of BCG-treated bladder cancer patients. Nevertheless, a larger patient cohort should be studied at this late time point in order to validate these observations, given the high rate of patient responses to BCG treatment, and the limited number of patients in this study. 

## 2. Results

### 2.1. Rationale for Study Design

**Figure 1:**
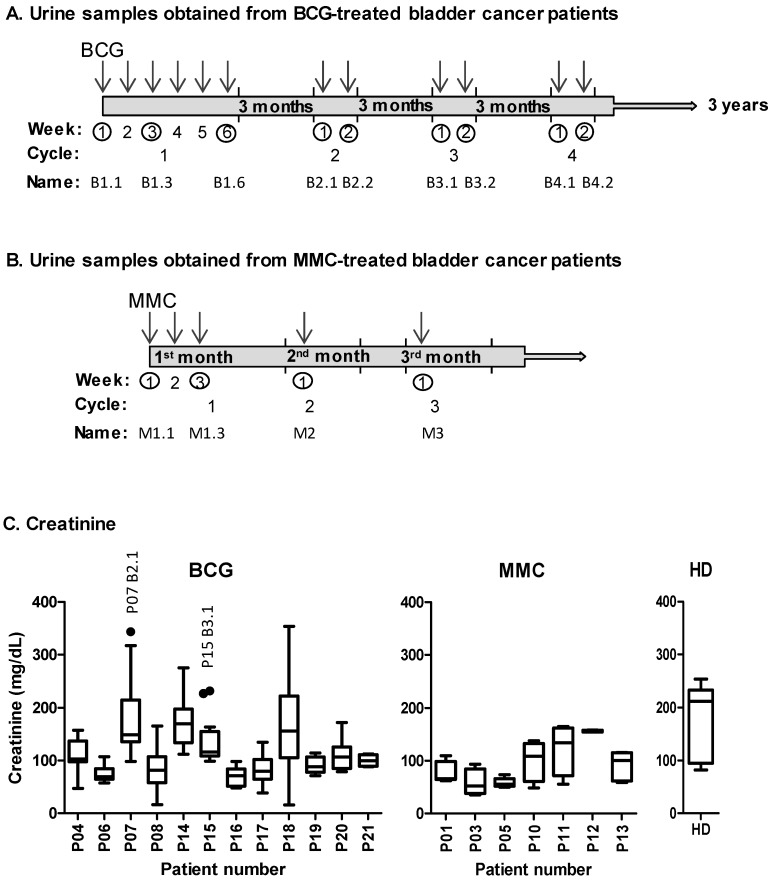
Schematic representations of the urine sample collection from non-muscle invasive bladder cancer (NMIBC) patients treated with *Bacille Calmette–Guérin* (BCG) and mitomycin C (MMC). (**A**) BCG. A cohort of NMIBC patients receiving intravesical BCG instillations (indicated by arrows) was recruited. During the first cycle, patients received one weekly instillation for six weeks. After a three-month rest, they received a second cycle of two weekly instillations, and this treatment continued for three years. The samples were obtained just before receiving the instillation at the time points indicated with a circle. The nomenclature used (B1.1, etc.) refers to the treatment (B for BCG), followed by two numbers corresponding to the cycle and the week of the instillation—for example, B1.3 means first cycle, third instillation. As each urine sample was collected before receiving an instillation, B1.1 corresponds to the basal conditions before BCG, while B1.3 corresponds to one week after the second instillation. Therefore, each sample was obtained either a week after the patient received an instillation, or after the three-month rest period, except for B1.1. (**B**) MMC. The samples from the control NMIBC patients receiving MMC instillations were collected during the first three months of treatment. Nomenclature corresponds to the first month and week, or the subsequent months. (**C**) Box and whiskers plot representing the concentration of creatinine in the urine of the BCG-treated patients, MMC-treated patients, and healthy donors (HD). At least nine samples per patient are included in the analysis, except for patients 19, 20, and 21, which include three to five samples. The outlier values were identified and are indicated by a dot. For the individual data of creatinine, see Supplementary Figure S1.

To test whether an efficient immune response to BCG could be evaluated in the urine samples collected seven days after BCG instillations, a broad study to screen for many different soluble factors along three years of treatment for each patient was planned. As, to our knowledge, no data were available in the literature on urinary cytokines after seven days of weekly instillations with BCG, each patient was followed for several cycles during treatment, including samples after the three-month rest periods. All of the patients were suffering from bladder cancer, and received intra-vesical instillations of either BCG or mitomycin C (MMC; Table 1). After the beginning of the study, two BCG-treated patients and one MMC patient were excluded because of clinical factors unrelated to the bladder cancer. Therefore, to have a more homogeneous cohort, the data included in the statistical analyses correspond to 12 BCG-treated patients. Multiple samples were obtained from each patient at different time points (nine samples from most of the 21 patients included in this study; fourteen BCG-treated and seven MMC-treated; Figure 1A). BCG treatment triggers an inflammatory and immune response, while MMC theoretically would not elicit this type of response, as it is directed against the cell proliferation of cancer cells. For this reason, MMC patients provide a good control, allowing monitoring the effect of bladder instillations in cytokine and chemokine release, as well as cancer-related inflammation events. The sample collection from MMC-treated patients followed a different calendar, because this treatment has a shorter course (Figure 1B). Tumor recurrence and adverse effects are presented in Table 2. Patient 8 (P8), had multiple recurrent T1G2 + CIS (carcinoma in situ) prior to this study, and, during this study, developed tumor recurrence and cancer progression. P7, P15, and P17 had recurrence; two of these patients (P7 and P17) also had BCG-treatment delay because of discomfort or cystitis. P17 abandoned the BCG-instillations late into the treatment (after the sixth instillation) because of BCG toxicity. 

### 2.2. NMIBC Patients Release Immune Mediators One Week after BCG Instillation

To evaluate how cytokine secretion varies over time after BCG instillations, a wide range of soluble factors were analyzed in the samples along the different cycles of instillations of a small cohort of BCG-treated NMIBC patients. The sample collection was done at diagnosis and seven days after the instillations; the first instillation of each cycle corresponds to the samples obtained after the rest periods, as marked in Figure 1A. Nine different time points, corresponding to the first year of BCG treatment, were included for statistical analysis; the control group of bladder cancer patients receiving MMC instillations was also analyzed (Figure 1B). As cytokines released into the urine are continuously depleted by micturition, any soluble factors detected must come from immune infiltration or deposition in the bladder on the day of collection (one week or three months after an encounter with the mycobacteria). Table 3 summarizes the panel of cytokines analyzed, and the number of patients releasing the indicated cytokine at least one time-point during the treatment. The value of the lower limit of detection for the test is also presented. As an internal control for the cytokine detection assays, the urine from three healthy donors was collected. As expected, these samples did not contain cytokines associated with the inflammatory processes. Seven days after instillation, a few soluble factors were found in the urine of the MMC-treated patients, except in P5, who had macroscopic hematuria. Thus, it seems reasonable to assume that the detection of urinary cytokines in BCG-treated patients is a manifestation of the immune response to the mycobacterial therapy. 

As the samples were obtained over a considerable time period, the content of the creatinine was also analyzed as a control for the variations in the concentration of urine taken at different moments (Figure 1C). The donor-to-donor variation was assessed and the patient samples outside the normal range were identified. Creatinine secretion is constant because of regulated kidney filtration, and is often used to normalize the protein content in 24-h urine samples. However, the urinary cytokines in the BCG-treated patients are not as a result of kidney function, but rather immune recruitment to the bladder urothelium, and they do not depend on urine concentration—outlier creatinine concentrations could be identified for certain donors, for example, P15, at cycle three, week one, the creatinine was higher than the other time points, suggesting concentrated urine on that day. However, it was possible to identify the samples with very low creatinine level but high amounts of cytokine, supporting the use of creatinine only as an indicator of sample-to-sample variation (Supplementary Figure S1). Therefore, the cytokine data are shown as absolute values rather than relative to the creatinine.

The Table 3 data reveal that the cytokine analysis in the urine from the BCG-treated NMIBC patients one-week post-instillation can provide information on the local immune response to the tumor after the acute, pro-inflammatory wave of cytokines has diminished.

### 2.3. CXCL10 (IP10) Is Highly Secreted in BCG-Treated NMIBC Patients Seven Days after the Instillations

**Figure 2:**
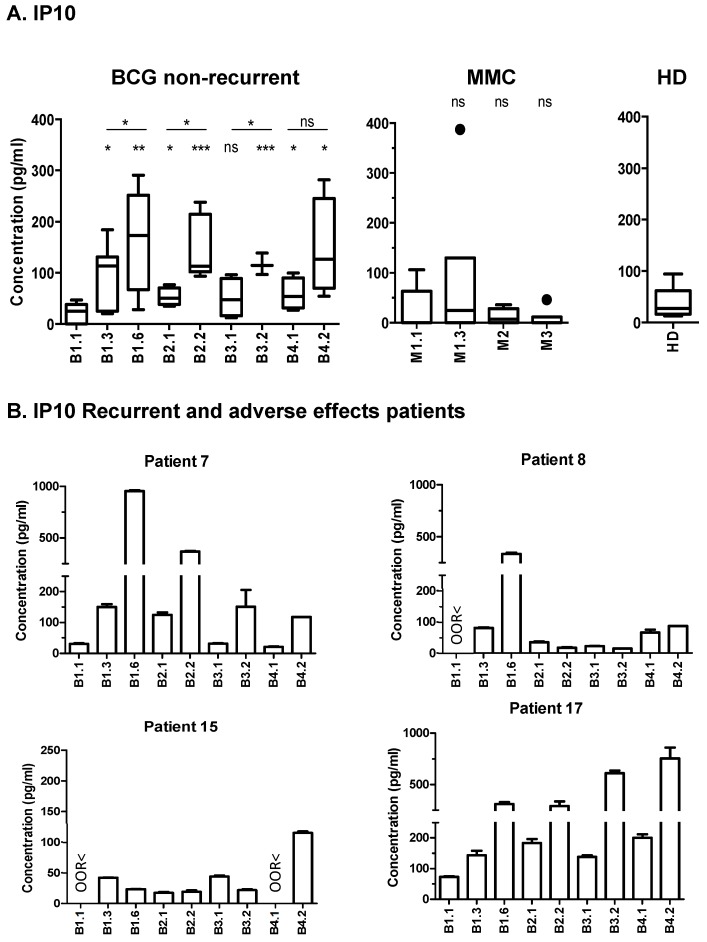
Detection of interferon-induced protein 10 (IP10 or CXCL10) in the urine from the non-muscle invasive bladder cancer (NMIBC) patients treated with either Bacille Calmette–Guérin (BCG) or mitomycin C (MMC). The urine from the patients receiving intravesical instillations of BCG (Ta/T1G3 or CIS, mean age of 68.5 years) or mitomycin C (MMC; Ta/T1G2, mean age of 67.4 years) was analyzed by Luminex, and is expressed in pg/mL. The samples were collected at different time points, as indicated in Figure 1, seven days after the indicated instillation. (**A**) Box and whiskers plot showing the statistics corresponding to the eight BCG-treated patients without recurrence, and the six MMC treated patients. The samples from three healthy donors (HD) were also analyzed as the internal control for the assay. The significance was analyzed by T-test, comparing all of the values with time zero (B1.1; lower raw of symbols), as well as each within each cycle (upper symbols); ns (non-significant) *p* > 0.05; * *p* < 0.05; ** *p* < 0.01; *** *p* < 0.001. (**B**) Individual data for the four BCG-treated patients that had either a recurrence or adverse effects. ORR< (out-of-range) indicates that the analyte concentration was below the limit of detection. Standard deviation (SD) corresponds to the experimental replicates.

Among all of the soluble factors tested, CXCL10 showed a remarkable pattern in the majority of the patients—absent at time 0, but strikingly increased during the first cycle of BCG instillations, reaching a maximum at the time of the sixth instillation. After the three-months resting period, the urinary CXCL10 levels had dropped, but again increased notably seven days after every recall instillation in the subsequent cycles. More than 50 pg/mL of CXCL10 were detected in the positive samples of 11 out of the 12 BCG-treated patients, as well as in two of the six MMC-treated patients (Table 3 and Figure 2A,B). The individual patient CXCL10 data, compared with the creatinine values, are depicted in Supplementary Figure S1. The graphs from the four BCG-treated patients that suffered either recurrence or progression (P7, P8, P15, and P17) are depicted separately from the other eight patients (Figure 2B). Two of these recurrent patients had problems tolerating BCG—P7 had to delay the continuation of BCG therapy after week six, which coincided with a much higher CXCL10 level (>800 pg/mL); similarly, P17 abandoned treatment after the sixth instillation, also because of BCG toxicity. Remarkably, the urinary CXCL10 in this patient also started to reach values above 800 pg/mL during the fourth cycle of instillations. In contrast, P15, a patient with an unusually low CXCL10 concentration, below 50 pg/mL in all of the urine samples collected, except cycle four, instillation two, showed no adverse reaction to BCG, but did suffer recurrence 2.4 years and 5 years after surgery (Figure 2B). P8, who also had tumor progression, also had a very low CXCL10 concentration in the urine samples after the first cycle of instillations.

The high amount of CXCL10 detected in the urine seven days after the instillations denotes a persistent production that occurs in the tumor environment, as a consequence of the BCG treatment. Because patients eliminate urine several times daily, the production of CXCL10 in the bladder of these patients is extremely high one week after the encounter with the mycobacteria. If we assume that a patient could urinate 800 mL to 2 L per day, a minimum of 200 ng of CXCL10 accumulated daily one week after the sixth instillation in the different patients (and up to 1.6 μg in the higher concentrations detected). This amount of cytokine is probably as a consequence of the immune response provoked by the vaccine, and it is worth trying to understand its role.

Although the number of patients studied is low, these data suggest that CXCL10 could be a marker of a good anti-tumoral immune response after BCG therapy, but that an excess of this factor might indicate that an excessive inflammatory response is occurring, leading to patient discomfort. 

**Figure 3:**
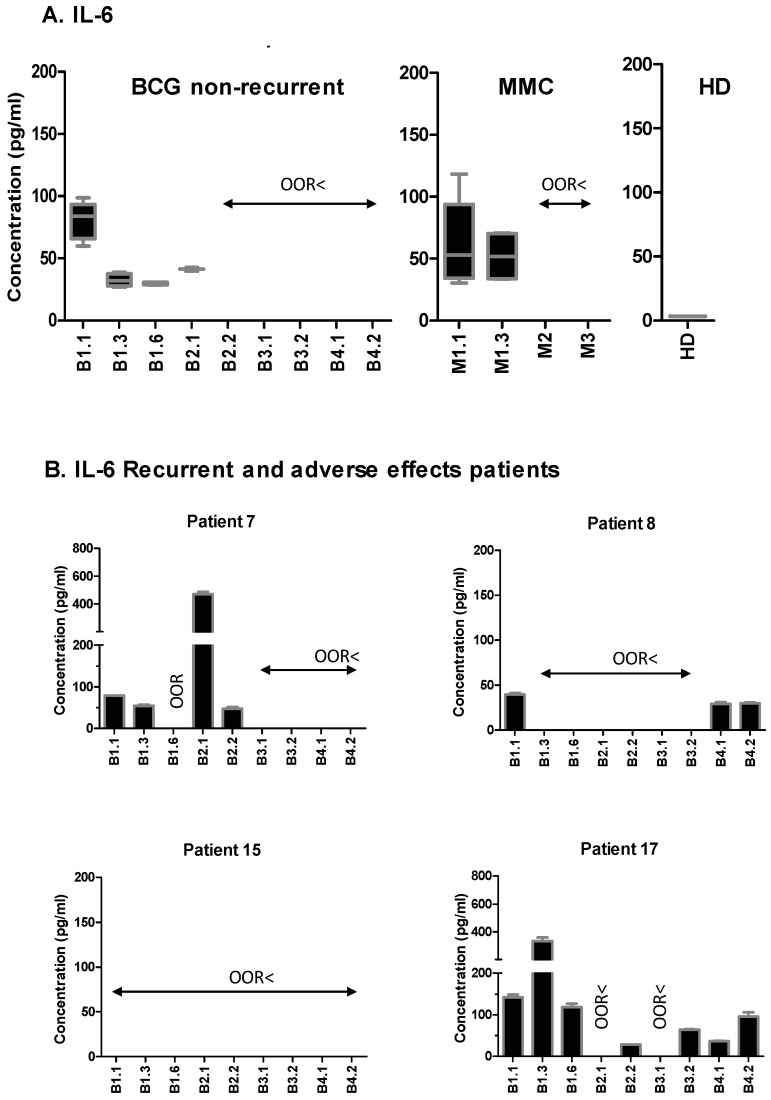
Detection of interleukin-6 (IL-6) in the urine from the non-muscle invasive bladder cancer (NMIBC) patients treated with either Bacille Calmette–Guérin (BCG) or mitomycin C (MMC). The urine from the same patients as in Figure 2 was analyzed by Luminex for its IL-6 content. The samples were collected at different time points of the treatment, as indicated in Figure 1. (**A**) Box and whiskers plot showing the statistics corresponding to the eight BCG-treated patients without recurrence, six MMC-treated patients, and three healthy donors (HD) for the internal control. (**B**) Individual data for the four BCG-treated patients that had recurrence. ORR< (out-of-range) indicates that the analyte concentration was below the limit of detection. Standard deviation (SD) corresponds to the experimental replicates.

**Figure 4:**
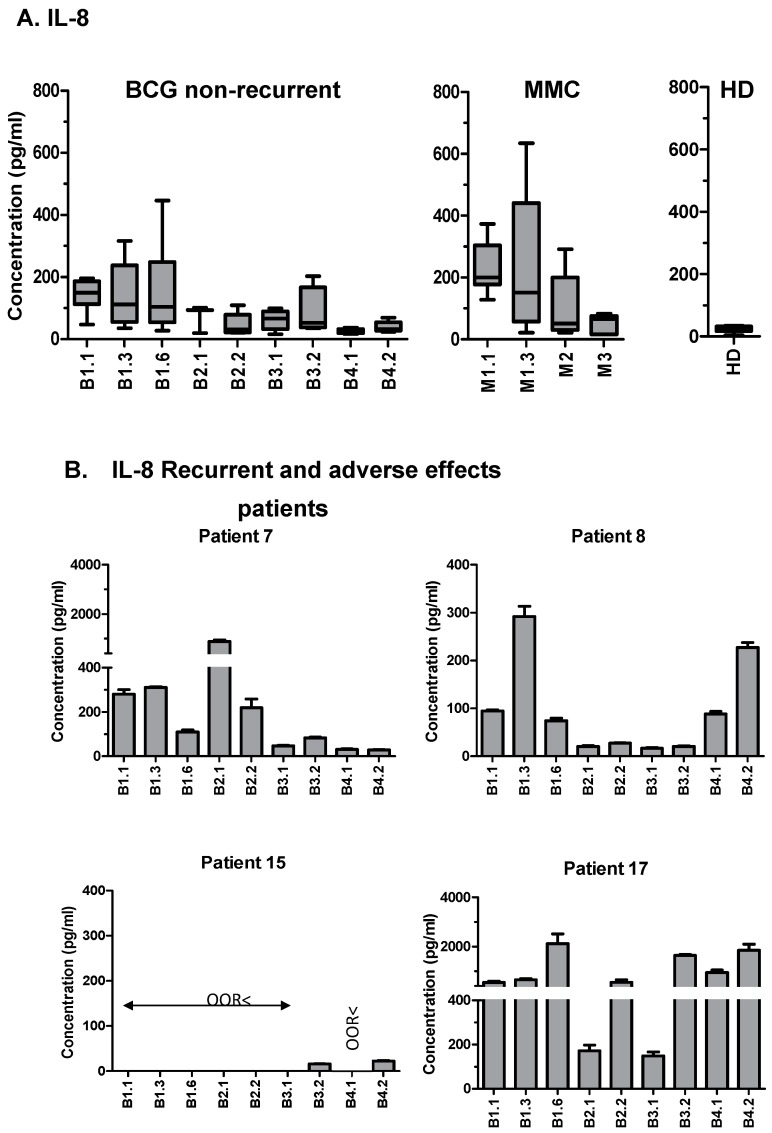
Detection of interleukin-8 (IL-8) in the urine from the non-muscle invasive bladder cancer (NMIBC) patients treated with either Bacille Calmette–Guérin (BCG) or mitomycin C (MMC). The urine from the same patients as in Figure 2 was analyzed by Luminex for its IL-8 content. The samples were collected at different time points of the treatment, as indicated in Figure 1. (**A**) Box and whiskers plot showing the values corresponding to the eight BCG-treated patients without recurrence, six MMC treated patients, and three healthy donors (HD) for internal control. (**B**) Individual data for the four BCG-treated patients that had recurrence. ORR< indicates that the analyte concentration was below the limit of detection. Standard deviation (SD) corresponds to the experimental replicates.

We also checked whether other cytokines were present in these patients, and compared the CXCL10 data for each one of them in relation to the other cytokines and the available clinical data. The adverse effects associated with the BCG treatment in P7 and P17 correlated with extremely high levels of CXCL10, and were accompanied by very high levels of IL-6 (>200 pg/mL) and IL-8 (>400 pg/mL), in contrast to the observed general downward trend for these cytokines in most patients (Figure 3 and Figure 4; Supplementary Figure S2). P7 had a delayed treatment because of discomfort, and an early recurrence (T1G2), seven months after the first transurethral resection (TUR). Strikingly, after the second TUR, the maintenance treatment of BCG was re-established and well tolerated. Consistent with our hypothesis, from that moment, the values for CXCL10 returned to the 50–200 pg/mL range, and the levels of IL-6 and IL-8 also decreased. This patient had no further recurrence in the next 5.7 years of follow-up.

Altogether, these data indicate that the determination of the urinary CXCL10, IL-6, and IL-8 levels seven-days after BCG instillations could help in the identification of BCG-responsive NMIBC patients, and that high concentrations of these cytokines might be associated with exacerbated immune responses. Studies with more patients are necessary in order to verify or reject this hypothesis, and thus we propose to start a multi-center study in a larger cohort of patients for the determination of these three factors, seven days after the instillations in the early cycles of the BCG-treatment of NMIBC patients.

### 2.4. Urinary CXCL10 Is Produced by Myeloid Cells 

**Figure 5:**
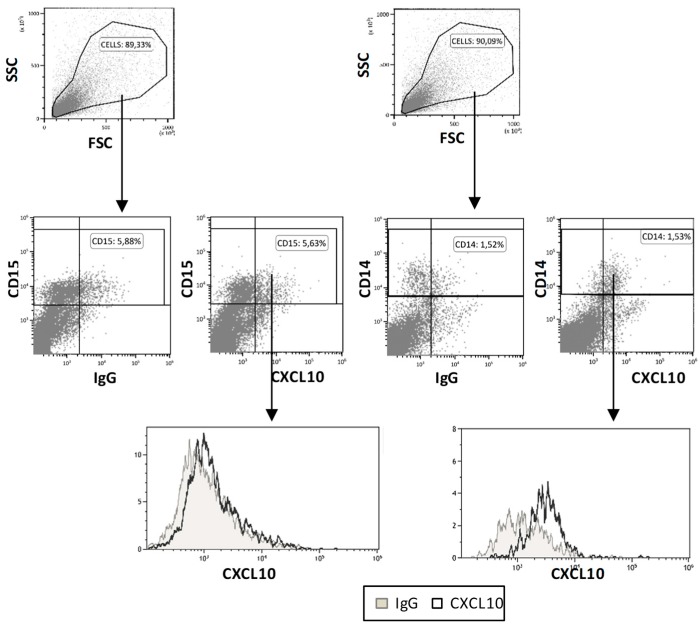
CXCL10 production: 12 urine samples from four BCG-treated bladder cancer patients were centrifuged to pellet cells. After washing, the cells were surface stained using CD14-APC and CD15-PB antibodies. For intracellular staining with either CXCL10-PE or immunoglobulin control (IgG-PE) antibodies, the cells were first fixed and permeabilized (see Methods). A representative experiment, with the gating strategy, is shown. CD14^+^ and CD15^+^ cells were gated for a comparison of the CXCL10 expression with the negative control (IgG-PE isotype) in the overlay histograms. CXCL10: C-X-C motif chemokine 10; APC: Allophycocyanin; PE: phycoerythrin.

To investigate the origin of CXCL10 in response to BCG, several urine samples of NIMBC patients were centrifuged to pellet cells, and intracellular staining flow cytometry was used to analyze the CXCL10 producer cells ex vivo. Several samples from four patients were analyzed (12 samples in total), and, in those samples with intracellular CXCL10 staining, the signal was detected in the CD14^+^ cells, but not in the CD15^+^ cells, confirming the role of myeloid cells in the BCG-mediated response against cancer secreting this chemokine (Figure 5).

### 2.5. CXCL10 Does Not Increase the Percentage of Anti-Tumour CD56^bright^ Cells, but Facilitates in the Migration of BCG-Activated Effector Cells

To further understand the role of the urinary CXCL10 found in bladder cancer patients treated with BCG, the activation and migration of the immune effector cells cultured in the presence of the chemokine were evaluated in vitro. As we recently published that a cytotoxic CD56^bright^ anti-tumor NK cell subpopulation was activated in the presence of BCG [6], and the CXCL10 blockade has been described to decrease the cytotoxic recognition of NK cells [14], we studied the effect of increasing the concentrations of CXCL10 on the activation of NK cells present in the peripheral blood mononuclear cells from healthy donors (Supplementary Figure S3). In vitro incubation with high concentrations of the chemokine for one week did not increase the percentage of the anti-tumor CD56^bright^ CD16^+^ NK cell subpopulation, and, in fact, higher concentrations seemed to decrease the number of these cells, suggesting that the generation of NK cells specialized in tumor recognition depends on the other cytokines generated during the initial cytokine cascades occurring in response to BCG.

**Figure 6:**
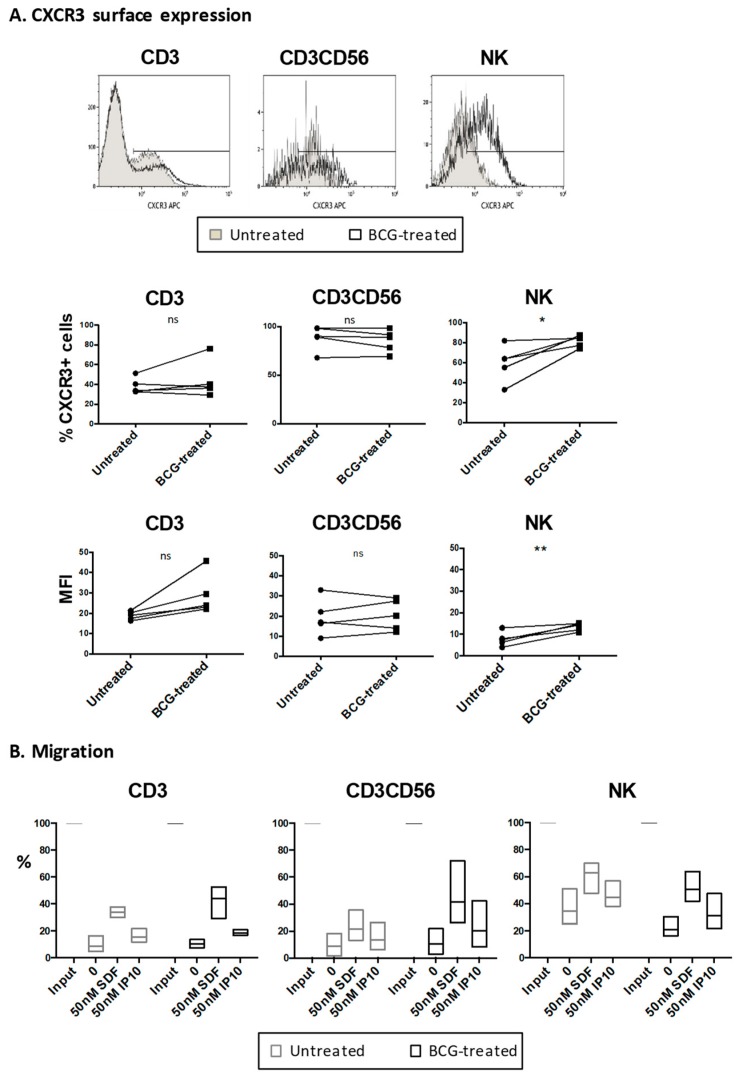
Effect of C-X-C motif chemokine 10 (CXCL10) on the surface expression of the CXCR3 receptor, and the migration of lymphocytes. Peripheral mononuclear blood cells (PBMCs) from healthy donors were incubated with or without Bacille Calmette–Guérin (BCG) at a 1:50 ratio (viable bacteria to PBMC). At day seven, the cells in suspension were recovered from the co-culture, centrifuged, and used for the experiments. (**A**) CXCR3 surface expression on different lymphocyte populations. Different lymphocyte populations were stained with the lineage markers, as indicated, in combination with CXCR3-Allophycocyanin (APC), and were analyzed by flow cytometry. Upper panels: histograms from one representative donor. Middle panels: T-test analysis comparing the percentage of CXCR3 positive cells between the untreated and BCG-treated cells, within each cell population, in five different donors (* *p* < 0.1). Bottom panels: T-test analysis comparing the mean fluorescence intensity (MFI) of CXCR3 between untreated and BCG-treated cells in the different cell populations, in five different donors (** *p* < 0.01). (**B**) Migration of lymphocyte populations towards CXCL10. The cells were placed on the top chamber of a transwell plate, and were allowed to migrate towards either the medium or medium supplemented with chemokines (50 nM SDF (Stromal cell-Derived Factor or CXCL12) as a positive control; 50 nM CXCL10). The graphs represent the percentage of migration of each cell population, relative to the total number of cells plated (input). The upper and lower quartiles are also shown. The statistical analysis performed was a two-way ANOVA (Analysis of Variance).

However, it was possible to detect higher amounts of the receptor for CXCL10 (CXCR3), on both CD56^bright^ and CD56^dim^ NK cells, and a subpopulation of CD3 T cells after the co-culture of PBMCs with BCG for one week (Figure 6A, Supplementary Figure S3). Although the percentage of CD3 cells expressing the CXCR3 receptor did not change in the presence of BCG, the mean fluorescence intensity (MFI) of the receptor was higher in the BCG-exposed T cells for each donor, however this difference was not statistically significant. The NK cells also increased the CXCR3 receptor after BCG exposure. In contrast, the effector CD3^+^CD56^+^ cells were positive for the receptor, independently of BCG exposure. This result implies that CXCL10 is more likely to participate in the recruitment of effector cells to the bladder, rather than their priming for immune response. To confirm this hypothesis, migration experiments were performed. The PBMCs from healthy donors were incubated on the upper chamber of a transwell. The percentage of cells that migrated towards the SDF (Stromal cell-Derived Factor or CXCL12); as a positive control), culture medium (as negative control), or CXCL10-containing medium, after 3 h of incubation, was measured by flow cytometry (Figure 6B). Lymphocytes remained in the upper chamber when no cytokine was included in the assay, and migrated strongly towards the SDF positive-control, and somewhat less towards CXCL10. The migration rate correlated with the expression of the CXCR3 receptor, and was independent of BCG activation. These results suggest that urinary CXCL10 will increase the attraction of the effector cells’, including CD3 and anti-tumor CD56^brigth^ NK cells, populations to the bladder in BCG-treated patients.

## 3. Discussion

To date, the only method to follow the response of NMIBC patients to BCG-treatment is regular urinary cytology combined with cystoscopy examinations. Other than the markers proposed from transcriptional studies [15,16], it has also been suggested that combinations of urinary cytokines could predict the response to BCG-therapy. However, the optimal combination of biomarkers is still not well defined. Here, for the first time, we analyzed the soluble factors present in the urine of a cohort of NMIBC patients seven days post-instillation. These data demonstrate that cytokine detection one week after contact with BCG is possible, and suggests that the determination of these soluble factors at this time-point could provide a simple and reliable method for monitoring the strength of the immune response triggered by BCG in NMIBC patients. In bladder cancer, urine composition reflects the tumor environment, and, as the bladder is emptied several times daily, our findings seven days post-instillations provide information on longer term immune responses compared with studies focusing on the urine collected only a few hours after BCG. The concentrations detected in urine seven days after the instillation with BCG imply the production of nanogram to microgram amounts of cytokine daily one week after receiving BCG by most of the patients, and variations in these amounts could be a consequence of the intensity of the immune response.

This pilot study was planned so as to analyze a large panel of cytokines and chemokines over time in a small cohort of NMIBC patients. The most striking result was the presence of considerable amounts of CXCL10 in urine samples one-week post-BCG instillation, following a similar pattern in most patients. However, high concentrations of CXCL10 (>250 pg/mL) were found in a small number of patients that had to interrupt BCG therapy, especially in those individuals that also had high levels of IL-6 and IL-8 (>200 pg/mL and >400 pg/mL, respectively), suggesting that high concentrations of CXCL10 might be a sign of an excessive inflammatory response, which is detrimental to the patients. In contrast, the few patients of this cohort with recurrence had no, or low, CXCL10 levels. In particular, in all of the samples obtained during the first year of instillations from P15, who had recurrence 2.5 years after TUR, only extremely low concentrations of CXCL10, IL-6, and IL-8 were detected.

CXCL10 has been detected in the urine of BCG-treated NMIBC patients previously. Specifically, in the urine collected either 12 h post-treatment during the first cycle of instillations [17], or collected 2 and 4 h after BCG instillations during weeks one and three [8]. In both cases, a significant increase of CXCL10 was observed. Bisiaux et al. also found increased levels of multiple pro-inflammatory cytokines in the urine collected 2–4 h after instillation at week three (mainly Macrophage inflammatory protein 1-beta (MIP-1β), IL-8, IL-6, IL-1β, Granulocyte Colony-Stimulating Factor (G-CSF), Granulocyte-Macrophage Colony-Stimulating Factor (GM-CSF), and Tumor Necrosis Factor (TNFα). It is interesting that several of these cytokines were also detected in our study at day seven post-instillation, but not in all of the cycles nor in all of the patients. This suggests that 2–4 h after the instillation, there is a very intense inflammatory response that probably specializes later the for elimination of the tumor. Another recent study described a panel of cytokines that could predict a response in 80% of the patients [12], analyzing the samples immediately before, and 4 h after BCG instillation at week-six, and immediately before and after the third instillation of the first maintenance course. This Cytokine Panel for Response to Intravesical Therapy (CyPRIT) demonstrates that a number of cytokines can be correlated with recurrence, including IL-6 and IL-8. However, CXCL10 was not included in this study. Although IL-8 was previously studied as a potential biomarker of responsiveness when released in the initial hours after instillation [18,19,20], our data show a decrease in this cytokine over time in responder patients. Based on our results, a focused study in a larger cohort is being planned in order to test this hypothesis and validate the use of CXCL10 as a biomarker for response.

We also studied whether CXCL10 could activate anti-tumoral NK cells. The importance of CXCL10 for NK recruitment and activation has been demonstrated in other systems [21], and stimulation via Toll-Like Receptor (TLR)4 or TLR9 could promote CXCL10-mediated NK recruitment in mice [22]. However, the data presented here suggest that a major role for CXCL10 would be in mediating the recruitment of effector cells towards the tumor site, and that BCG can facilitate the upregulation of the CXCR3 receptor in this context. Thus, our data reveal the importance of the CXCL10/CXCR3 axis in this therapy, and complements other studies in the models of checkpoint inhibitor immunotherapies [23]. 

The biological role of CXCL10 is the chemoattraction of immune cells, promotion of T cell adhesion to endothelial cells, and inhibition of bone marrow colony formation and angiogenesis, in response to interferon gamma (IFNγ). CXCL10 is secreted by several cell types (including monocytes, endothelial cells, and fibroblasts), and attracts granulocytes and effector leukocytes to the bladder for tumor elimination. Indeed, CXCL9 and CXCL10 production has been shown to lead to CXCR3-dependent recruitment of murine NK cells into solid tumors for immune elimination [24,25]. The data presented here demonstrate that myeloid cells can produce CXCL10 in BCG-treated patients. Thus, new questions arise from our data, namely: “Are there any other cells responsible for producing CXCL10?”, “What cells are recruited into the bladder in response to CXCL10?”, and “What soluble factors are directly involved in the secretion of CXCL10 and/or other related soluble factors?”. CXCL10 is often induced by interferon, and although IFNγ per se was undetectable in the urine collected one week post-BCG, this cytokine may be produced earlier in order to shape the sustained innate immune response. Thus, CXCL10 could be part of a wave of myeloid-derived factors contributing to a sustained immune response to the tumor.

## 4. Materials and Methods 

### 4.1. Patients

A prospective study was designed where one group of NMIBC patients was treated with bladder instillations of BCG, while the second group received mitomycin-C (MMC). The MMC-treated patients were included as the control, because they also received intra-vesical instillations. These patients would not be expected to undergo the same inflammatory or immune responses as the BCG-treated patients, and so served as a control for cancer-related inflammation not due to BCG therapy. The experiments were conducted with the understanding and written informed consent of each participant. Approval by the Institutional Review Board, as well as local and regional ethical committees (CEI La Paz Hospital HULP-1067 8th February 2011; revised by CEI Infanta Sofía Hospital and CSIC Local Ethical Committee) were obtained, and the study conformed to the principles expressed in the Declaration of Helsinki. Patient enrolment occurred between 2012 and 2016. 

Patients with biopsy-proven, completely resected solitary or multiple Ta-T1G3 (1973 World Health Organization (WHO) classification) urothelial carcinoma of the bladder and/or CIS were included in the first group, and received BCG treatment—weekly instillations of the BCG TICE strain (one vial) for six weeks (induction period), followed by two weekly instillations every three months for three years (maintenance). Patients with biopsy-proven, completely resected solitary or multiple Ta–T1, grade 2 (1973 WHO classification) urothelial carcinoma of the bladder, were included in the control group, and were treated with MMC. Patients on the MMC treatment received one weekly instillation of 40 mg for four weeks (induction period), and afterwards, one instillation every month for 6 to 12 months (maintenance). The BCG vaccination status of the patients was unknown. All of the T1G3 patients had TUR, but not the TaG3 nor CIS patients. 

The urine was obtained before each instillation, at different times during treatment at Hospital Infanta Sofía (Madrid, Spain), and the patients were followed for an average of 4.7 years (Figure 1). As a result of logistic problems, in a few cases, samples could not be obtained from patients at every single time-point. For example, as it is clear from the figures, only three to five urine samples could be analysed from P19, P20, and P21, although they received the same treatment and their clinical outcome was also followed for several years. The clinico-pathological data are summarised in Table 1. Tumour recurrence was defined as a newly identified bladder tumour relapse after a previous negative follow-up cystoscopy, either systemic or local, and with or without muscle infiltration. Patients with a positive cytology were not considered as recurrence initially; they were sent for cystoscopy and biopsy to confirm or exclude tumour presence. Papillary tumours were considered recurrence. Cancer progression was defined as tumour relapse with bladder muscle infiltration and/or metastasis. The cut-off date for the last follow-up was October 2017. 

### 4.2. Sample Handling

Spontaneous urine samples were obtained at the clinic immediately prior to BCG or MMC instillation. Within 2 h, the samples were centrifuged at 400× *g* to remove crystals and separate the cells that were further washed with PBS (Phosphate Buffered Saline). The clarified urine samples were stored at −80 °C in aliquots until analysis, and were thawed once for each determination.

### 4.3. Creatinine Determination

The urinary creatinine concentration was determined using the creatinine colorimetric detection kit (Enzo Life Sciences; ADI-907-030A, Farmingdale, New York, USA), according to the manufacturers’ instructions. Samples were diluted 1:20 with distilled/deionized water, and analyzed in duplicate. Four-parameter logistic standard curves were generated, and the creatinine concentrations within each urine sample were interpolated using GraphPad Prism version 5.00 (GraphPad Software, USA, www.graphpad.com). Healthy donors had a mean creatinine concentration of 186 mg/dL.

### 4.4. Cytokine Measurements

The urinary cytokine determinations were performed blind, without knowing the outcome of each patient. As an internal control for the assays, the urine from three healthy donors (two age-matched males and one non-age-matched female) was collected. The urine samples were diluted 1:2 using Calibrator Diluent RD6-52 (R&D Systems, Minneapolis, Minnesota, USA) plus Tris-HCl pH 7.6 (final concentration of 10 mM). The diluted patient urine samples were analyzed in duplicate using human magnetic Luminex^®^ screening assays (R&D Systems, Minneapolis, Minnesota, USA) and Luminex^®^ 100™ or 200™ Luminex analyzer instruments (Qiagen, Germantown, Maryland, USA), according to the manufacturers’ instructions. Five-parameter logistic standard curves were generated, and the analyte concentrations within each sample were interpolated using the Bio-Plex Manager™ Software (Bio-Rad, Hercules, California, USA).

### 4.5. Statistical Analyses

GraphPad Prism version 5.00 (GraphPad Software, USA, www.graphpad.com) and Microsoft Excel were used. In the box and whiskers plots, the ends of the boxes are the upper and lower quartiles, and the median is marked by a horizontal line inside the box. The whiskers extend to the highest and lowest observations. The outliers were identified by the Tukey method. For the Luminex analysis, the plots were generated from the data within the limits of detection. So, the absence of data means that the cytokine or chemokine was undetectable in those samples.

### 4.6. IP10 Stimulation of PBMCs

The PBMCs from healthy donors (Regional Transfusion Centre, Madrid, Spain) were isolated by centrifugation on Ficoll-Hypaque (GE Healthcare, Boston, Massachusetts, USA), and cultured for one week in RPMI-1640 (Biowest, Nuaillé, France) supplemented with 5% Fetal Bovine Serum (FBS), 5% human male AB serum, 2 mM glutamine, 1 mM sodium pyruvate, 0.1 mM non-essential amino acids, 10 mM Hepes, 100 U/mL penicillin, and 100 U/mL streptomycin (Biowest, Nuaillé, France) at a concentration of 1 × 10^6^ cells/mL in 96-well plates with rhCXCL10 (ImmunoTools, Friesoythe, Germany) at 1–10 ng/mL. At day seven, the cells were recovered for extracellular staining.

### 4.7. Flow Cytometry

For the flow cytometry, the cells were washed with PBA (PBS supplemented with 0.5% bovine serum albumin (BSA), 1% FBS, and 0.1% sodium azide), and incubated with conjugated antibodies against surface markers (CD3-CF (Immunostep, Salamanca, Spain); CD56-PC5 (Phycoerythrincyanin 5), CD16-PE-Cy7 (phycoerythrin-Cy7), CD14-APC (Allophycocyanin), CD15-PB (Pacific Blue), and CXCR3-APC (BioLegend, San Diego, California, USA) at 4 °C for 30 minutes in the dark. For intracellular staining, after surface labelling, the cells were fixed with 1% p-formaldehyde for 10 min at room temperature (RT), permeabilized with 0.2% saponin for 10 min at RT, and stained with anti-CXCL10-PE (BD, San Jose, California, USA) or mouse IgG2a-PE (Beckman Coulter, Brea, California, USA) for 30 min. The cells were washed in PBA and analyzed using a Gallios Flow Cytometer (Beckman Coulter, Brea, California, USA). The analysis of the experiments was performed using either Kaluza (Beckman Coulter, Brea, California, USA) or FlowJo software (Ashland, Oregon, USA, https://www.flowjo.com).

### 4.8. Peripheral Blood Mononuclear Cell Stimulation with BCG

BCG Tice strain (from Merck Sharp and Dohme, Kenilworth, New Jersey, USA) was used. The aliquots of the reconstituted BCGs were prepared in an RPMI 10% dimethyl sulfoxide (DMSO), and stored at −20 °C. For the BCG stimulation, experiments were performed as previously described [6]. Briefly, 10^6^ PBMCs/mL were incubated in 24-well plates with or without BCG at a 1:10 ratio (viable bacteria: PBMC). One week later, the cells in suspension were recovered from the co-culture, and were centrifuged and analyzed by flow cytometry to identify the NK and CD3 populations, as explained above.

### 4.9. Migration Experiments

The bottom chamber of a transwell with a 50-µm pore size was filled with 600 μL of a medium (RPMI, 0.1% BSA 10mM Hepes pH 7.5), containing either no cytokine, 50 mM SDF (CXCL12) as a positive control, or 50 mM CXCL10 (IP10). The PBMCs were placed in 100 μL of medium at the top chamber, and incubated for 3 h at 37 °C. The cells that migrated towards the bottom chamber were collected and analyzed by flow cytometry. 

## 5. Conclusions

In summary, we demonstrate here a prolonged production of CXCL10 in cancer patients that respond effectively after receiving BCG instillations, and we propose that this in vivo immune response could provide a new tool to measure the strength of the BCG-stimulated immune response against cancer in patients. The assay of the other cytokines, notably IL-6 and IL-8, in the urine of the NMIBC patients could also be important in order to evaluate the immune response presumably involved in the prevention and clearance of the tumor. Sample collection seven days after BCG instillations has the advantage of being much more convenient for patients, as it does not involve more visits to the clinic. A larger cohort of patients should now be studied in order to definitively establish the cytokines that could be combined at early time points, for a reliable correlation to the BCG anti-tumor response.

## Figures and Tables

**Table 1:** Patient clinico-pathological description.

		BCG		MMC	
		N	%	N	%
Gender	Male	10	83.3	4	66.7
	Female	2	16.7	2	33.3
Age	60 or less	2	16.7	0	0.0
	61–70	6	50.0	5	83.3
	more than 71	4	33.3	1	16.7
Size	<3 cm	8	66.7	2	33.3
	≥3 cm	3	25.0	4	66.7
	N/A	1	8.3		
Number	<3	7	58.3	5	83.3
	≥3	4	33.3	1	16.7
	N/A	1	8.3		
Primary		11	91.7	6	100.0
Recurrent		1	8.3	0	0.0
CIS		5	41.7		
T1		7	58.3	4	66.7
Ta		3	25.0	2	33.3
G1		0	0.0	1	16.7
G2		1	8.3	5	83.3
G3		10	83.3	0	0.0
Treatment delayed as a result of side effects		4	33.3	0	0.0
Stop as a result of side effects		1	8.3	0	0.0
Recurrence—*n* (%)		4	33.3	0	0.0
Progression		1	8.3	0	0.0

BCG—Bacille Calmette–Guérin; MMC—mitomycin C; CIS—carcinoma in situ; N/A- non-applicable.

**Table 2:** Recurrence and progression events in the cohort of BCG-treated patients.

Patient	Tumor	Stop/Delay	Recurrence	Progression
P7	Primary	After B1.6 as a result of discomfort	After B1.6, TUR Restart BCG with delay	
P8	Previous tumors in 1990, 1995			2.7 years after B1.1 T4a
P15	Primary	Delay after B3.2 as a result of prostate treatment	2.4 years from B1.1: 2 × T1G2; 5 years from B1.1: 3 × TxGx	
P17	Previous TaG2	After B6.1 as a result of cystitis	3.1 years from B1.1 TxGx	

TUR—transurethral resection; BCG—Bacille Calmette–Guérin.

**Table 3:** Summary of cytokines and chemokines detected in urine.

Cytokine/Chemokine	Limit * (pg/mL)	BCG ^#^	Mitomycin ^#^	Healthy
IL-1β	10.0	3/12	-	-
IL-2	28.03	5/12	4/6	-
IL-4	16.87	-	-	-
IL-5	14.59	-	-	-
IL-6	20.08	6/12	3/6	-
IL-8	17.74	7/12	5/6	-
IL-9	2414.91	-	-	-
IL-10	14.81	-	-	-
IL-12p70	249.14	-	-	-
IL-15	8.18	-	-	-
IL-17	18.64	-	-	-
IL-18BPa	32.55	+	+	+
IL-22	17.49	2/12	-	+
IL-23	293.58	1/12	-	-
TNF-α	19.75	-	-	-
IFN-γ	6.3	-	-	-
MIP1β	48.23	-	-	-
MIG	843.21	-	-	-
CXCL10	3.54	11/12	2/6	-
RANTES	14.53	5/12	1/6	-
TRAIL	34.98	+	+	+

* Lower detection limit for each cytokine. ^#^ Number of patients with cytokine concentration in the urine above the limit of detection, at at least one time-point + or–means that all of the patients or healthy donors were positive or negative (i.e., below or above the limits of detection), respectively. IL—interleukin; TNF—Tumor Necrosis Factor; IFN—interferon; MIP—Macrophage Inflammatory Protein; MIG—Monokine Induced by Gamma interferon; CXCL10—Interferon-induced Protein 10 (IP10); RANTES—Regulated on Activation, Normal T-cell Expressed and Secreted; TRAIL—TNF-Related Apoptosis-Inducing Ligand.

## Supplementary Materials

The following are available online at https://www.mdpi.com/2072-6694/11/7/940/s1, Figure S1: Comparison of Creatinine and CXCL10 Concentrations in Urine from NMIBC Patients Treated with either BCG or MMC; Figure S2: Comparison of IL-6 and IL-8 Detected in the Urine of BCG-treated and MMC-treated patients; Figure S3: Effect of CXCL10 on NK Cell Activation.
