# Supplementary material for: BCG Therapy of Bladder Cancer Stimulates a Prolonged Release of the Chemoattractant CXCL10 (IP10) in Patient Urine
Source: Cancers (Basel). 2019 Jul 4;11(7):940. doi: 10.3390/cancers11070940 (PMC6678801; doi:10.3390/cancers11070940)
Supplement: Supplementary file 1 [file cancers-11-00940-s001.pdf]

Supplementary Materials

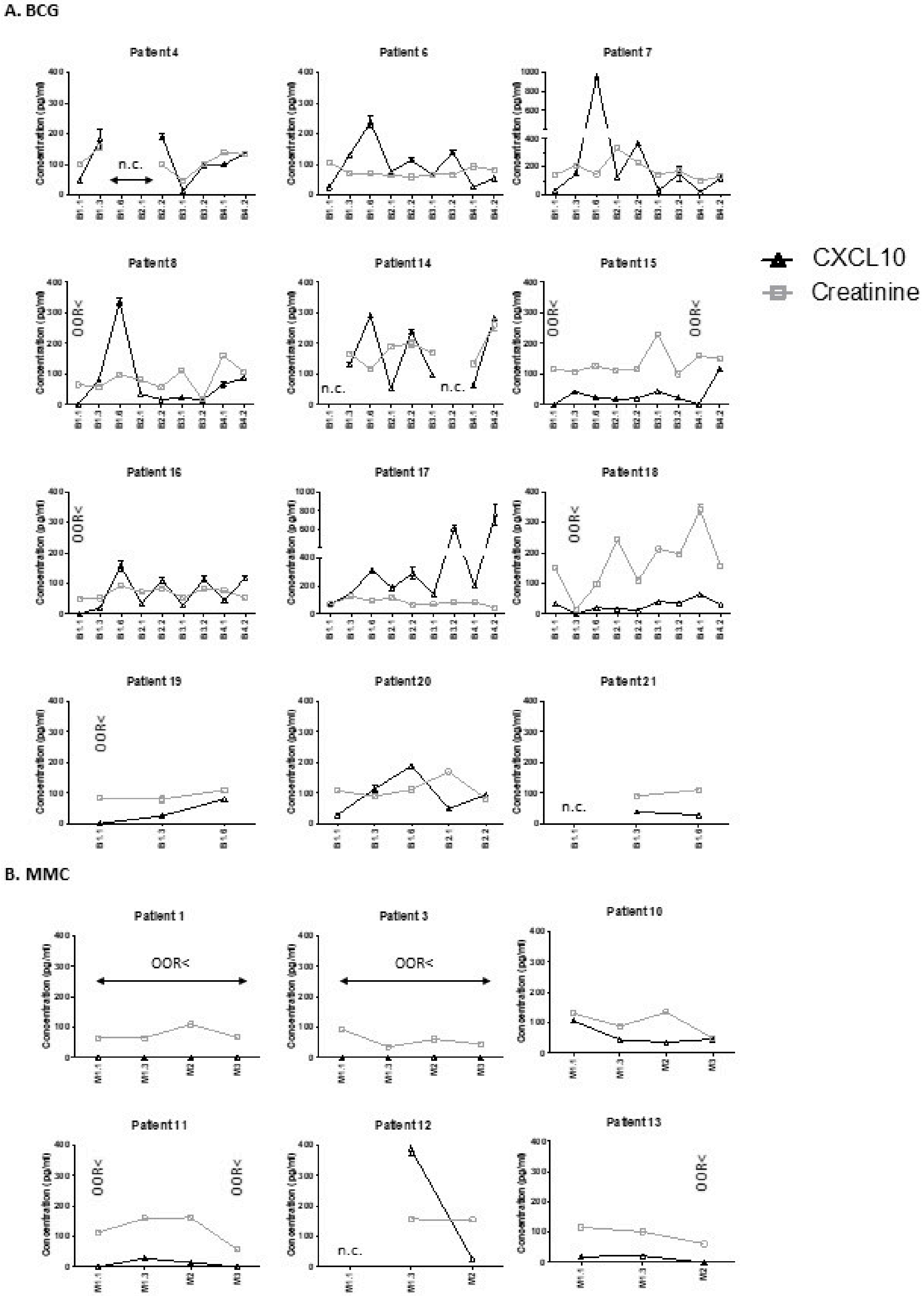

C. BCG

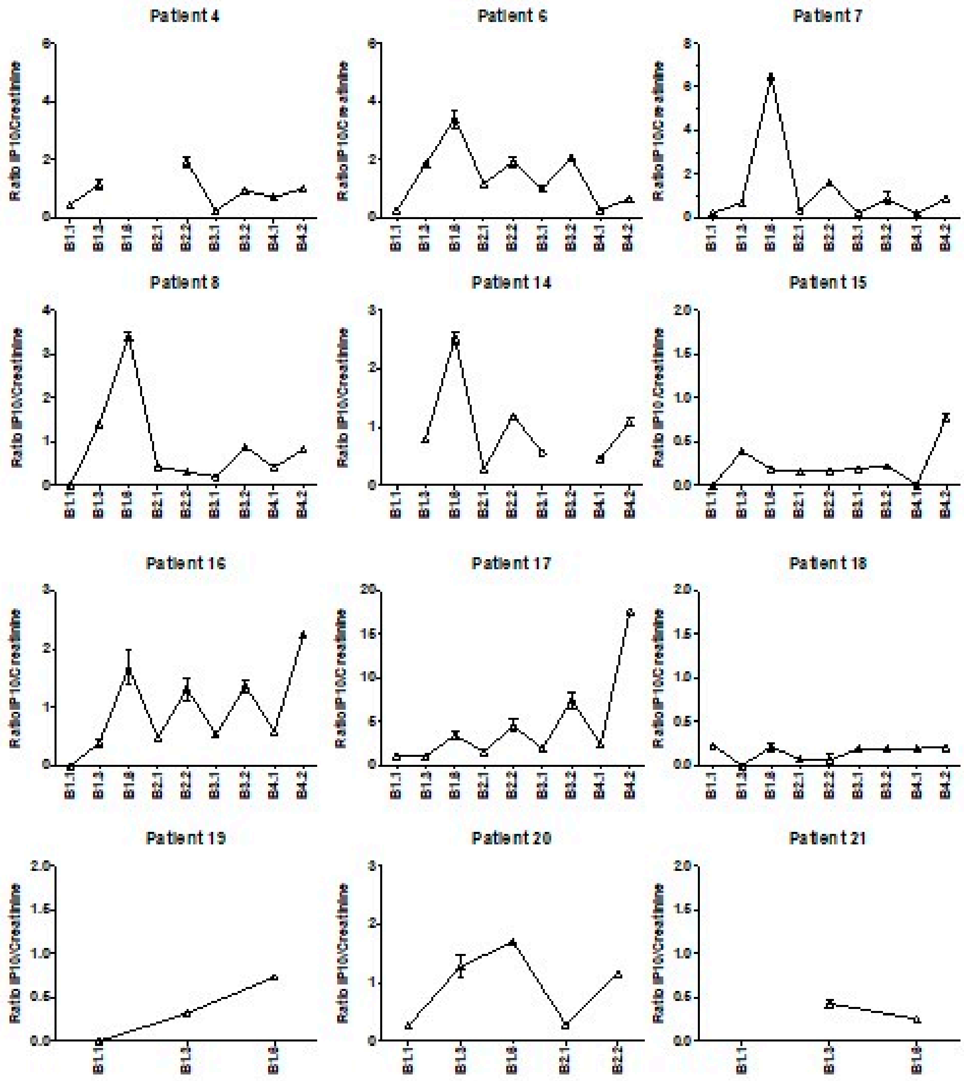

D. MMC

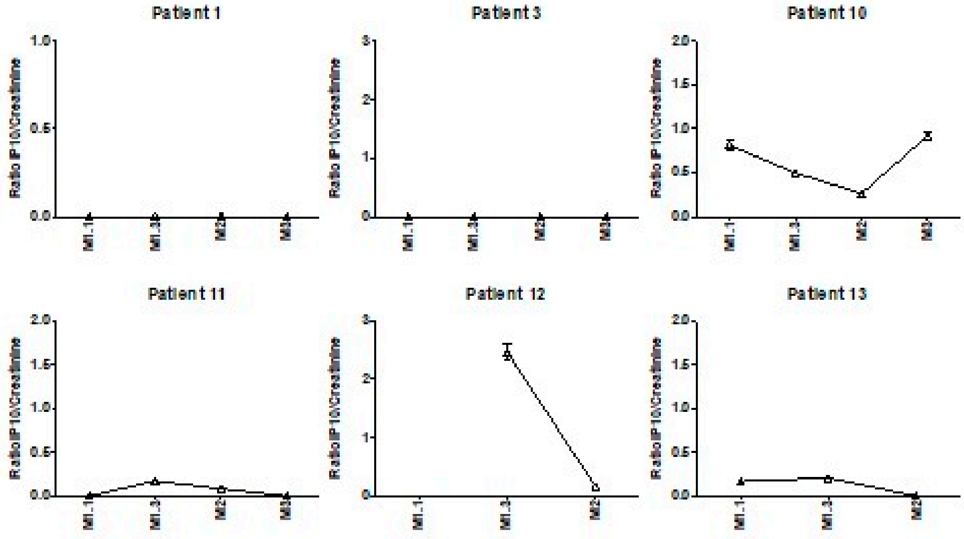

Figure S1. Comparison of creatinine and CXCL10 concentrations in urine from NMIBC patients treated with either BCG or MMC. Urine from NMIBC patients receiving intravesical instillations of BCG (A) or MMC (B) was analysed for the content of CXCL10 by Luminex and creatinine by ELISA.

Graphs show different time point samples for each patient analysed in duplicate (bars represent the range of the measurement). ORR< (Out-of-Range) indicates that the CXCL10 concentration obtained was below the limit of detection for CXCL10. n.d. means not determined because the sample was not available. (C, D) Concentration of CXCL10 relative to creatinine content in urine.

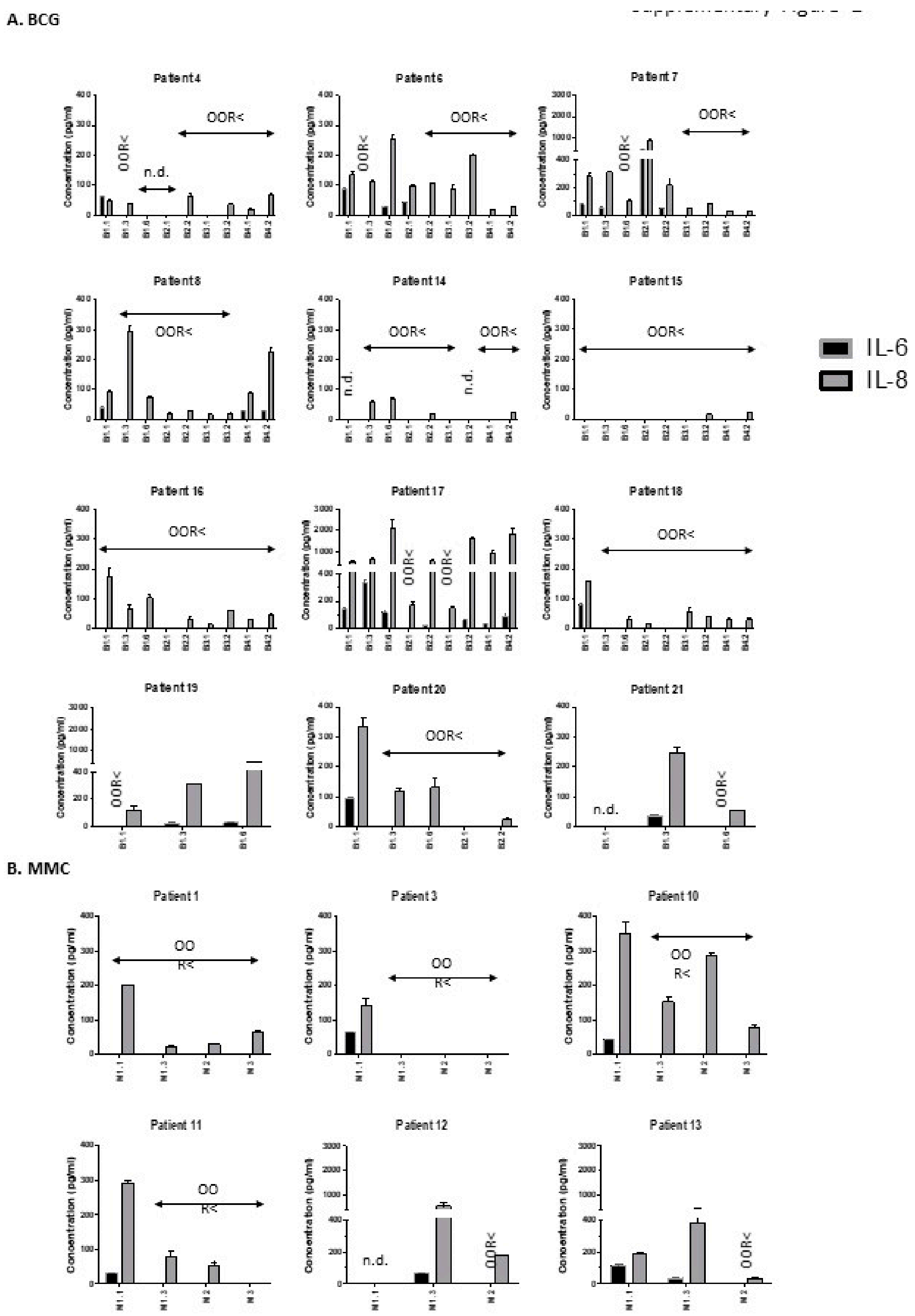

Figure S2. Comparison of IL-6 and IL-8 detected in the urine of BCG-treated and MMC-treated patients. (A) Different panels show the individual data of the IL-6 and IL-8 content by Luminex in the urine of BCG (A) and MMC (B)-treated patients. Graphs show different time point samples for each patient. ORR< indicates that the value obtained was below the limit of detection. n.d. means not determined because the sample was not available.

A. CD56 expression after CXCL10 incubation

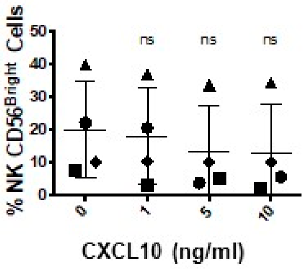

B. CXCR3 expression after one week in culture with BCG

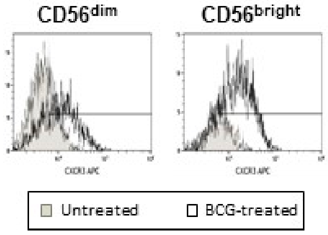

Figure S3. Effect of CXCL10 on NK cell activation. (A) PBMCs from 4 healthy donors were incubated with or without increasing concentrations of CXCL10 as indicated. At day 7, cells in suspension were recovered, centrifuged and analysed by flow cytometry. The graph represents the percentage of $CD56^{bright}$ cells obtained for each donor within the NK cell gate ($CD3^{-}CD56^{+}$) for a given concentration of chemokine. Non-significant (ns) changes between donors were obtained when analysing data using one-way ANOVA. Different symbols represent different donors. (B) Effect of CXCL10 on surface expression of CXCR3 receptor. PBMCs from healthy donors were incubated with or without BCG at a 1:50 ratio (viable bacteria to PBMC). At day 7, cells in suspension were recovered from the co-culture, centrifuged and stained with CD3 CD56, in combination with CXCR3-APC and analysed by flow cytometry within the NK cell population gate. Histograms from one representative donor out of 5 different donors are shown.
